# Supplementary material for: Ochratoxin A-Induced Hepatotoxicity through Phase I and Phase II Reactions Regulated by AhR in Liver Cells
Source: Toxins (Basel). 2019 Jun 29;11(7):377. doi: 10.3390/toxins11070377 (PMC6669489; doi:10.3390/toxins11070377)
Supplement: Supplementary file 1 [file toxins-11-00377-s001.pdf]

Supplementary Materials: Ochratoxin A-Induced Hepatotoxicity through Phase I and Phase II Reactions Regulated by AhR in Liver Cells

Hye Soo Shin, Hyun Jung Lee, Min Cheol Pyo, Dojin Ryu and Kwang-Won Lee

Table S1. The human and mouse qPCR primer sequences used in the experiments.

**Human qPCR primer**	**Forward (5'-3')**	**Reverse (5'-3')**
AhR	TGG ACA AGG AAT TGA AGA AGC	AAA GGA GAG TTT TCT GGA GGAA
PXR	CAT GAG GGG GGT AGC AAA GC	TGC AGG GGA TCT CCC TCT TC
Nrf2	AAC CAG TGG ATC TGC CAA CTA CTC	CTG CGC CAA AAG CTG CAT
CYP1A1	CAC CAT CCC CCA CAG CAC	ACA AAG ACA CAA CGC CCC TT
CYP1A2	TCA TCC TGG AGA CCT TCC GAC A	GCC ACT GGT TTA CGA AGA CAC AG
CYP3A4	TGT GTT GGT GAG AAA TCT GAG G	CTG TAG GCC CCA AAG ACG
HO-1	CTT CTT CAC CTT CCC CAA CA	AGC TCC TGC AAC TCC TCA AA
GCLC	AGT TGA GGC CAA CAT GCG AA	TGA AGC GAG GGT GCT TGT TT
Caspase 3	GGA AGC GAA TCA ATG GAC TCT GG	GCA TCG ACA TCT GTA CCA GAC C
Caspase 9	GTT TGA GGA CCT TCG ACC AGC T	CAA CGT ACC AGG AGC CAC TCT T
Bax	TCA GGA TGC GTC CAC CAA GAA G	TGT GTC CAC GGC GGC AAT CAT C
GAPDH	TCA CCA CCA TGG AGA AGG C	GCT AAG CAG TTG GTG GTG CA
**Mouse qPCR primer**	**Forward (5'-3')**	**Reverse (5'-3')**
CYP1A1	CAT CAC AGA CAG CCT CAT TGA GC	CTC CAC GAG ATA GCA GTT GTG AC
CYP1A2	CAT CAC AAG TGC CCT GTT CAA GC	AAT GCT CCA GGT GAT GGC TGT G
HO-1	GTG ATG GAG CGT CCA CAG C	TGG TGG CCT CCT TCA AGG
GCLC	AAG GCG TGT TTC CTG GAC TCA T	TGT CTT GCT TGT AGT CAG GAT GGT T
GAPDH	CCA ATG TGT CCG TCG TGG ATC T	GTT GAA GTC GCA GGA GAC AAC C
